# Suppression of Hepatic Bile Acid Synthesis by a non-tumorigenic FGF19 analogue Protects Mice from Fibrosis and Hepatocarcinogenesis

**DOI:** 10.1038/s41598-018-35496-z

**Published:** 2018-11-21

**Authors:** Raffaella Maria Gadaleta, Natasha Scialpi, Claudia Peres, Marica Cariello, Brian Ko, Jian Luo, Emanuele Porru, Aldo Roda, Carlo Sabbà, Antonio Moschetta

**Affiliations:** 1 Department of Interdisciplinary Medicine, “Aldo Moro” University of Bari, Bari, Italy; 2 INBB, National Institute for Biostructures and Biosystems, Rome, Italy; 3 NGM Biopharmaceuticals, South San Francisco, CA USA; 4 Department of Chemistry “Giacomo Ciamician”, Alma Mater Studiorum, University of Bologna, Bologna, Italy; 5 National Cancer Center, IRCCS Istituto Tumori “Giovanni Paolo II”, Bari, Italy

## Abstract

Critical regulation of bile acid (BA) pool size and composition occurs via an intensive molecular crosstalk between the liver and gut, orchestrated by the combined actions of the nuclear Farnesoid X receptor (FXR) and the enterokine fibroblast growth factor 19 (FGF19) with the final aim of reducing hepatic BA synthesis in a negative feedback fashion. Disruption of BA homeostasis with increased hepatic BA toxic levels leads to higher incidence of hepatocellular carcinoma (HCC). While native FGF19 has anti-cholestatic and anti-fibrotic activity in the liver, it retains peculiar pro-tumorigenic actions. Thus, novel analogues have been generated to avoid tumorigenic capacity and maintain BA metabolic action. Here, using BA related *Abcb4*^−/−^ and *Fxr*^−/−^ mouse models of spontaneous hepatic fibrosis and HCC, we explored the role of a novel engineered variant of FGF19 protein, called FGF19-M52, which fully retains BA regulatory activity but is devoid of the pro-tumoral activity. Expression of the BA synthesis rate-limiting enzyme *Cyp7a1* is reduced in FGF19-M52-treated mice compared to the GFP-treated control group with consequent reduction of BA pool and hepatic concentration. Treatment with the non-tumorigenic FGF19-M52 strongly protects *Abcb4*^−/−^ and *Fxr*^−/−^ mice from spontaneous hepatic fibrosis, cellular proliferation and HCC formation in terms of tumor number and size, with significant reduction of biochemical parameters of liver damage and reduced expression of several genes driving the proliferative and inflammatory hepatic scenario. Our data *bona fide* suggest the therapeutic potential of targeting the FXR-FGF19 axis to reduce hepatic BA synthesis in the control of BA-associated risk of fibrosis and hepatocarcinoma development.

## Introduction

Hepatocellular carcinoma (HCC) is the sixth most common malignancy and the third most frequent cause of cancer-related death^1^. The lack of effective therapeutic options makes the quest for novel putative treatment strategies of paramount importance. The gut-liver axis homeostasis relies on a tight control of bile acid (BA) levels in order to avoid BA overload that is critical in the pathogenesis of hepatic diseases.

BAs are the end products of cholesterol catabolism, synthesized in the liver and released into the small intestine after meal ingestion. BA production and circulation are tightly regulated via the nuclear receptor, farnesoid X receptor (FXR). In the liver, FXR reduces conversion of cholesterol to BAs by downregulating the rate limiting enzyme of BA synthesis cytochrome P450 A1 (CYP7A1), via the small heterodimer partner (SHP). Moreover, FXR promotes hepatic bile secretion by increasing the expression of crucial BA transporters. In the enterocytes, BA-bound FXR induces the transcription of the fibroblast growth factor FGF15/19 (mouse and human, respectively), an enterokine secreted into the portal circulation, able to reach the liver and bind to the fibroblast growth factor receptor 4 (FGFR4)/β-Klotho complex. This initiates a phosphorylation cascade in the c-jun N-terminal kinase-dependent pathway, ultimately inhibiting CYP7A1 expression hence BA synthesis^2^, a mechanism working in synergy with the hepatic FXR-SHP-dependent one^3^.

Altered BA signaling in the liver and intestine is associated with severe diseases including the development of cholestasis and HCC^4–8^. Hepatic diseases causing intrahepatic cholestasis, such as the progressive familial intrahepatic cholestasis type 2 and 3 (PFIC2-3) caused by the multidrug resistance protein 3 (MDR3) deficiency, represent a specific risk of HCC development, especially in children^9^. *ATP-binding cassette transporter* (*Abcb4*)^−/−^ and *Fxr*^−/−^ mice are commonly used as elective models of HCC development^8,10–15^. *Abcb4*^−/−^ mice lack the liver-specific permeability-glycoprotein responsible for phosphatidylcholine flippase on the outer leaflet of the hepatocyte canalicular membrane and therefore for secretion of phosphatidylcholine in bile. The absence of phospholipids from bile causes bile regurgitation into the portal tracts^16^, inducing accumulation of toxic BA levels, and consequent fibrosis that leads to hepatocyte dysplasia first and HCC at 12–15 months of age, mimicking human progressive familial intrahepatic cholestasis^17^. *Fxr*^−/−^ mice exhibit increased BA pool size and display cell hyperproliferation leading to the development of spontaneous HCC at 12 months of age^18^.

Strategies aimed at limiting BA overload are anticipated to provide hepatoprotection, as earlier reported in *Fxr*^−/−^ mice treated with BA-sequestering agents^8^. We have recently shown that specific intestinal Fxr activation is sufficient to restore BA homeostasis in *Fxr*^−/−^ mice, thus protecting them from age-related hepatic inflammation, fibrosis, and cancer^18^. Also, we have recently shown that long-term administration of a Fxr agonist enriched diet prevents spontaneous hepatocarcinogenesis in *Abcb4*^−/−^ mice via Fxr-Fgf15-dependent suppression of hepatic *Cyp7a1*^19^.

The discovery of the role of FXR target gene, FGF15/19, in the feedback regulation of hepatic BA synthesis shed light on the physiological relevance of the crosstalk between the liver and intestine in the context of BA homeostasis^2,20,21^. FGF19 has also been implicated in HCC development. In fact, it is amplified in HCC and its expression is induced in liver of patients with extrahepatic cholestasis^22–24^. Interestingly, induction of *Fgf15* expression in mice by intestinal Fxr overexpression protects against cholestasis and fibrosis, along with a reduction of the BA pool size^25^ suggesting that modulation of FGF19 levels could offer benefits in a plethora of BA-related metabolic disorders. However, despite its protective action, FGF19 has been shown to be protumorigenic and accelerate hepatic tumour formation in *Abcb4*^−/−^ mice in an FGFR4-dependent fashion^26,27^ raising doubts on the safety of a chronic administration of this hormone^28^. Recently, the generation of a FGF19 variant (M70) that is equally effective as endogenous Fgf15/19 in terms of bile acid metabolic regulatory actions but does not show any pro-tumorigenic activity in 8 months old *Abcb4*^−/−^ mice^27^ was described. Moreover, administration of FGF19-M70 in healthy human volunteers potently reduces BA synthesis^29^. This data provided us with the impetus to investigate the potential protective role of a novel non-tumorigenic FGF19 variant in spontaneous HCC development during BA dysregulation.

In the present work, we show for the first time that the non-tumorigenic variant of FGF19, namely FGF19-M52, retaining its intrinsic metabolic effects on *Cyp7a1* repression and consequent reduction of hepatic BA synthesis, protects *Abcb4*^−/−^ and *Fxr*^−/−^ mice against spontaneous hepatocarcinogenesis thus electing hepatic BA suppression as a metabolic strategy to prevent fibrosis and HCC in susceptible models.

## Results

### M52 is a non-tumorigenic variant of FGF19 that retains activity in regulating BA synthesis

**Figure 1:**
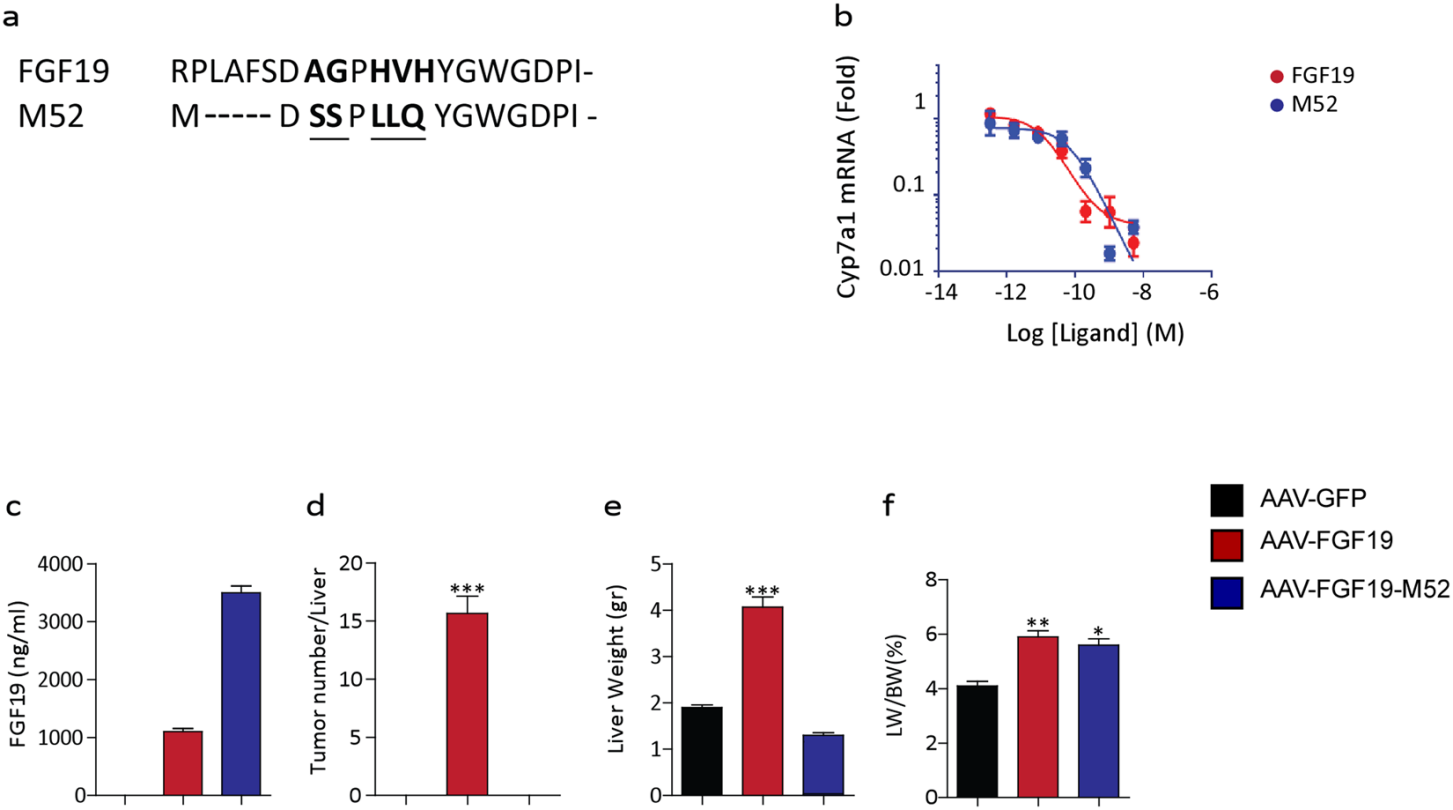
M52 is a non-tumorigenic variant of FGF19 that retains activity in regulating BA synthesis. (**a**) Alignment of protein sequences of M52 and FGF19 in the N-terminal region. Mutations introduced into M52 are underlined. (**b**) Repression of CYP7A1 mRNA expression by AAV-M52 vs AAV-GFP in primary human hepatocytes. (**c**) Plasma levels of transgene expression, (**d**) number of tumors per liver, (**e**) liver weight and (**f**) percentage of liver weight over body weight ratio in db/db mice injected with AAV-GFP (black bar), AAV-FGF19 (red bar) or AAV-FGF19-M52 (blue bar). All values represent means ± SEM. Statistical significance comparing either FGF19 or M52 versus control (*p < 0.05, **p < 0.01, ***p < 0.001) assessed by one-way ANOVA followed by Dunnett’s post hoc test.

The novel engineered variant of the FGF19 protein M52 has been recently generated. M52 differs from wild-type FGF19 by five amino acid substitutions (A30S, G31S, H33L, V34L, H35Q) and five–amino acid deletion at the N terminus (Fig. 1a). In order to characterize the M52 variant and compare it to the full length FGF19, we tested CYP7A1 repression in primary human hepatocytes. qRT-PCR analysis shows that relative *mRNA* levels of CYP7A1 do not change in both FGF19 and M52 cell treatment, indicating that the M52 variant retains his biological activity of repression of *de novo* BA synthesis (Fig. 1b). Further *in vivo* analysis in *db/db* mice revealed that M52 is present in plasma as well as FGF19 (Fig. 1c). Moreover, while FGF19 administration causes an increased number of tumors per liver as well as a raised liver weight and liver/body weight ratio compared to controls, the M52 variant does not show any tumorigenic activity (Fig. 1d–f, respectively).

### FGF19-M52 metabolically protects Abcb4^−/−^ mice from age-related liver damage

**Figure 2:**
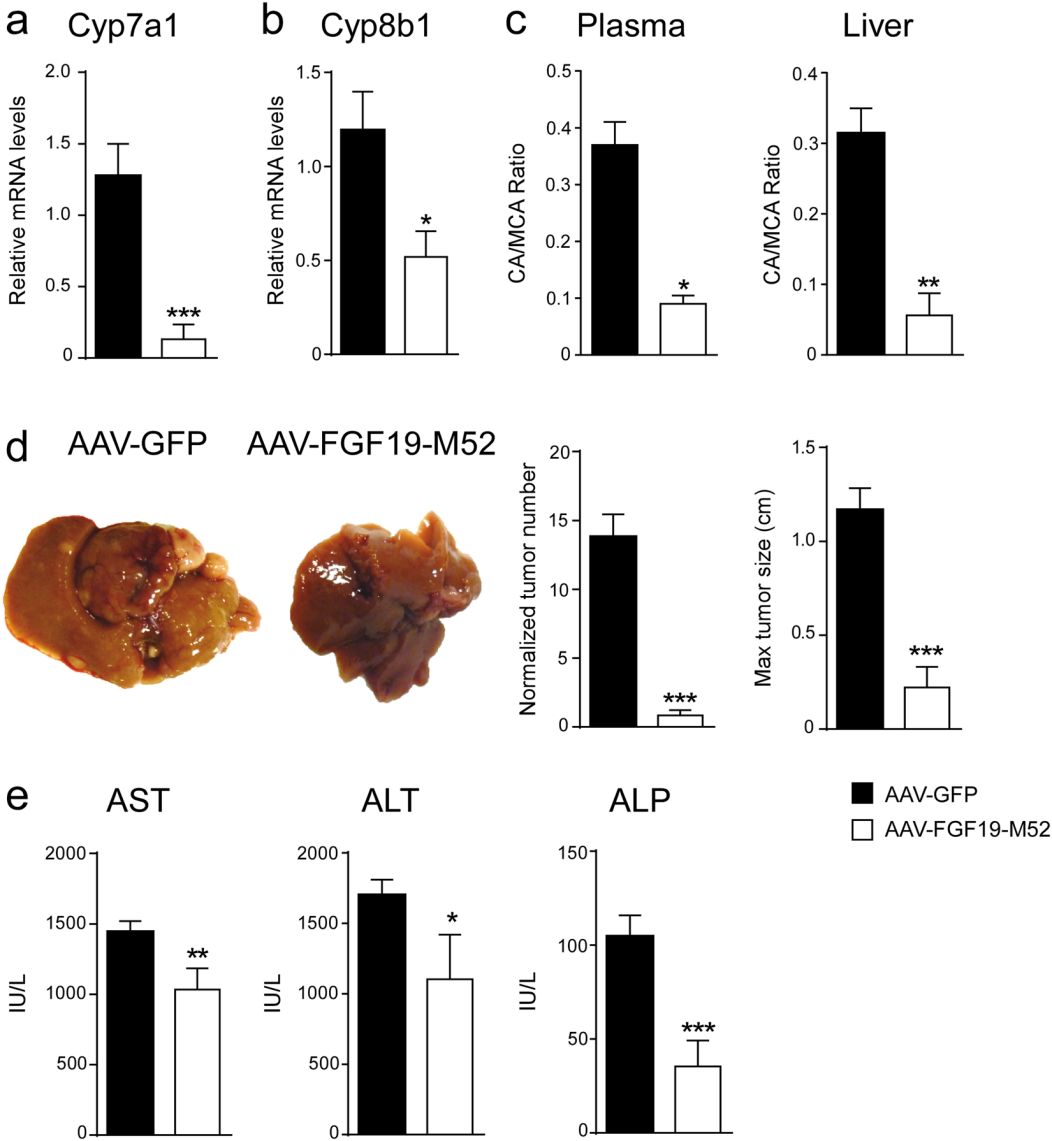
FGF19-M52 analogue protects Abcb4^−/−^ mice from age-related liver damage and HCC progression. qPCR of hepatic (**a**) *Cyp7a1* (**b**) *Cyp8b1*. Expression was normalized to *Cyclophillin*. (**c**) BA composition (CA/MCA Ratio) in murine plasma and liver. (**d**) Macroscopic appearance of livers, normalized tumor number and maximum tumor size of 16-months-old *Abcb4*^−/−^ mice (n = 14–24). (**e**) Biochemical parameters of liver damage in 16 months old *Abcb4*^−/−^ mice. Statistical significance (*p < 0.05, **p < 0.01, ***p < 0.001) was assessed by Mann-Whitney’s U test.

The non-tumorigenicity of the novel FGF19-M52 variant and its concomitant ability to maintain CYP7A1 repression, prompt us to explore its metabolic and antitumoral ability in aged *Abcb4*^−/−^ mice, a murine model of impaired BA homeostasis-induced HCC. Aged *Abcb4*^−/−^ mice have been elected as a unique animal model for studying HCC pathogenesis because they resemble many features of human HCC progression. 100% of aging *Abcb4*^−/−^ mice display metabolic derangement due to liver inflammation and toxicity induced by a progressive increase and accumulation of BAs. ELISA measurement shows a strikingly higher amount of FGF19 in adenovirus (AAV)-FGF19-M52-treated mice compared to AAV-green fluorescent protein (GFP) controls (AAV-GFP 8.38 ± 6.28 vs AAV-FGF19-M52 434.5 ± 83.65 pg/ml). Compared to the control group, AAV-FGF19-M52-injected mice displayed a significant lower hepatic mRNA expression of the key limiting enzyme of BA synthesis *Cyp7a1* (Fig. 2a). Also, the *cytochrome P450 family 8 subfamily B member 1* (*Cyp8b1*) - another critical enzyme controlling the ration between Cholic Acid (CA) and Chenodeoxyxholic acid (CDCA) by regulating the synthesis of CA - results inhibited in AAV-FGF19-M52-treated mice compared to control (Fig. 2b). These changes were translated in an extremely powerful reduction of the plasmatic total BA pool size (AAV-GFP 74.65 ± 12.54 vs AAV-FGF19-M52 0.63 ± 0.11 μM) and a shift in both plasma and liver BA composition to a more hydrophilic BA pool profile due to the enrichment in muricholic acid (MCA) (Fig. 2c, Tables 1 and 2).

**Table 1:** Serum Bile Acid Composition.

Genotype	CA%	UDCA%	CDCA%	DCA%	LCA%	MCA%	TCA%	TUDCA%	TCDCA%	TDCA%	TLCA%	TMCA%
*Abcb4*^−/−^-GFP	0.45 ± 0.04	0.23 ± 0.02	0.12 ± 0.01	0.12 ± 0.03	0.03 ± 0.01	5.42 ± 1.15	24.46 ± 1.58	2.08 ± 0.73	3.22 ± 0.13	0.17 ± 0.03	0 ± 0	63.69 ± 1.24
*Abcb4*^−/−^-M52	4.98 ± 1.2	0.02 ± 0.01	5.8 ± 1.65	0.03 ± 0.02	2.6 ± 0.6	18.32 ± 3.81	4.92 ± 3.32	0.12 ± 0.1	0.69 ± 0.61	0.03 ± 0.02	0 ± 0	62.63 ± 2.73
*Fxr*^−/−^-GFP	12.5 ± 3.17	0.83 ± 0.25	1.25 ± 0.38	8.2 ± 1.64	0.2 ± 0.06	8.1 ± 1.24	47.81 ± 3.69	0.68 ± 0.166	0.68 ± 0.166	3.78 ± 0.67	0 ± 0	15.93 ± 2.29
*Fxr*^−/−^-M52	11.76 ± 3.03	1.22 ± 0.27	0.56 ± 0.14	3.22 ± 0.94	0.15 ± 0.05	28.88 ± 8.29	23.9 ± 6.86	0.53 ± 0.12	0.08 ± 0.04	0.36 ± 0.13	0 ± 0	29.37 ± 9.5

CA, cholic acid; UDCA, ursodeoxycholic acid**;** CDCA, chenodeoxycholic acid; DCA, deoxycholic acid; LCA, lithocholic acid; MCA, muricholic acids; TCA, taurocholic acid; TUDCA, tauroursodeoxycholic acid; TCDCA, taurinechenodeoxycholic acid; TDCA, taurinedeoxycholic acid; TLCA, taurinelithocholic acid; TMCA, taurinemuricholic acids.

**Table 2:** Liver Bile Acid Composition.

Genotype	CA%	UDCA%	CDCA%	DCA%	LCA%	MCA%	TCA%	TUDCA%	TCDCA%	TDCA%	TLCA%	TMCA%
*Abcb4*^−/−^-GFP	1.23 ± 0.27	0.36 ± 0.03	0.5 ± 0.09	0.01 ± 0.01	0.02 ± 0	34.73 ± 3.77	21.31 ± 1.69	0.88 ± 0.27	2.99 ± 0.25	0.18 ± 0.05	0.05 ± 0.02	37.74 ± 5.06
*Abcb4*^−/−^-M52	0.7 ± 0.45	0.82 ± 0.15	0.96 ± 0.11	0 ± 0	0.06 ± 0.02	70.04 ± 4.6	4.02 ± 1.86	0.19 ± 0.05	2.15 ± 0.26	0.04 ± 0.04	0 ± 0	21.02 ± 3.82
*Fxr*^−/−^-GFP	5.31 ± 0.24	1.15 ± 0.25	0.78 ± 0.18	0.56 ± 0.12	0.12 ± 0.03	25.98 ± 1.61	52.59 ± 2.88	0.23 ± 0.08	1.57 ± 0.34	8.62 ± 1.54	0.27 ± 0.09	2.83 ± 0.42
*Fxr*^−/−^-M52	1.28 ± 0.24	0.17 ± 0.04	0.14 ± 0.02	0.07 ± 0.02	0.02 ± 0	16.02 ± 3.9	24.96 ± 4.79	0.68 ± 0.1	0.3 ± 0.04	0.82 ± 0.22	0.05 ± 0.01	55.5 ± 4.09

CA, cholic acid; UDCA, ursodeoxycholic acid**;** CDCA, chenodeoxycholic acid; DCA, deoxycholic acid; LCA, lithocholic acid; MCA, muricholic acids; TCA, taurocholic acid; TUDCA, tauroursodeoxycholic acid; TCDCA, taurinechenodeoxycholic acid; TDCA, taurinedeoxycholic acid; TLCA, taurinelithocholic acid; TMCA, taurinemuricholic acids.

**Figure 3:**
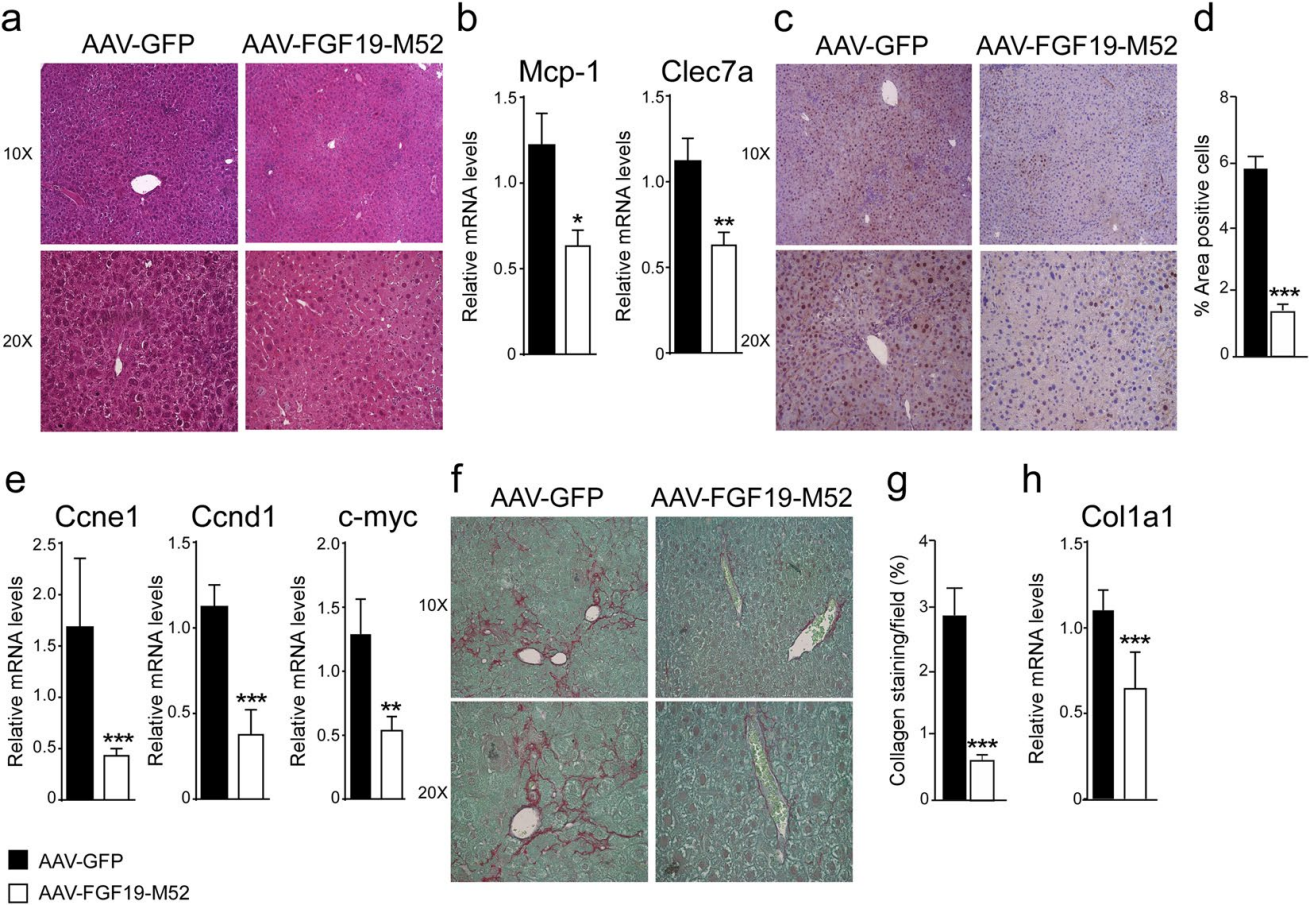
FGF19-M52 analogue protects Abcb4^−/−^ mice from fibrosis and cellular proliferation. (**a**) Liver histology assessed by hematoxylin and eosin (H&E) staining and observed by light microscopy (magnification, 10–20X). (**b**) *Mcp-1* and *Clec7a* gene expression levels assessed by real time qPCR in tumor-free liver extracts. (**c**) Hepatic immunohistochemical staining of the oncogene Ccnd1. (**d**) Quantification of Ccnd1 staining assessed with ImageJ and expressed as % of the area occupied by positive cells. (**e**) *CCnd1*, *Ccne1* and *c-myc* gene expression levels assessed by real time qPCR in tumor-free liver extracts. Expression was normalized to *Cyclophillin*. (**f**) Hepatic collagen deposition assessed by Sirius Red staining (magnification, 10–20X). (**g**) Quantification of collagen deposition assessed with ImageJ and expressed as % of collagen staining/field (**h**) *Col1a1* gene expression levels assessed by real time qPCR in tumor-free liver extracts. Expression was normalized to *Cyclophillin*. All values represent mean ± SEM. Statistical significance (**p < 0.01, ***p < 0.001) was assessed by Mann-Whitney’s U test.

In order to investigate whether the metabolic changes induced by the FGF19-M52 analogue would translate in protection from HCC occurrence, macroscopic analysis of 16 months old *Abcb4*^−/−^ mice liver was performed at the day of sacrifice. Earlier studies suggested that attenuation of systemic BA overload reduce number and size of liver tumors^8,18^. As expected, 100% of *Abcb4*^−/−^ mice injected with the control adenovirus AAV-GFP showed macroscopically identifiable tumours. Remarkably, almost no tumors could be identified in *Abcb4*^−/−^ mice injected with AAV-FGF19-M52 compared to controls (Fig. 2d), and when a tumor was borne in these mice it was significantly smaller compared to control mice. This protection was accompanied by a great reduction of plasma biochemical parameters of liver damage, as indicated by a decrease of the hepatic enzymes aspartate transaminase (AST), alanine aminotransferase (ALT) and alkaline phosphatase (ALP) in FGF19-M52 mice compared to GFP-treated mice (Fig. 2e). We also analysed liver morphology and HE staining shows liver injury and disrupted hepatic parenchymal structure in GFP-injected *Abcb4*^−/−^ mice, while FGF19-M52-injected mice display a more preserved hepatic parenchyma and less inflammatory infiltrates (Fig. 3a). This is accompanied by a reduction of the immune response regulators *C-type lectin domain family 7 member A* (*Clec7a*) and *monocyte chemo-attractant protein 1* (*MCP-1*) (Fig. 3b).

### FGF19-M52 protects Abcb4^−/−^ mice from hepatic collagen deposition and fibrosis

Liver fibrosis results from chronic damage to the liver in conjunction with the accumulation of extracellular matrix (ECM) proteins, which is a characteristic of most types of chronic liver diseases^30^. Lack of the *Abcb4* gene elicits a plethora of detrimental cell responses, including hepatic fibrosis, thus laying the foundation to hepatocarcinogenesis. In order to explore the effect of our FGF19 analogue on fibrogenesis we examine the extent of hepatic collagen deposition in liver isolated from aged *Abcb4*^−/−^ mice injected with or without M52. Liver immunohistochemical analysis with Sirius Red staining and quantification of the signal revealed that livers from M52-treated *Abcb4*^−/−^ mice were less fibrotic as compared to GFP mice (Fig. 3f,g). This finding was paralleled by *mRNA* inhibition of *Collagen type 1 alpha 1* (*Col1a1*) in M52 *Abcb4*^−/−^ mice compared to the control group (Fig. 3h).

### FGF19-M52 protects Abcb4^−/−^ mice from overexpression of HCC oncogenes

Alterations of cell-cycle-related genes have been documented in hepatocarcinogenesis^31,32^ as well as a compensatory proliferative response to BA-induced hepatocellular damage, thus providing evidence for a prognostic role of G1-S modulators in HCC. *Abcb4*^−/−^ mice also present with cell hyperproliferation. *CyclinD1* (*Ccnd1*) is a key regulator of cell cycle progression, and its overexpression has been reported to be sufficient to initiate hepatocellular carcinogenesis^33^. Accordingly, mouse models of disrupted BA homeostasis, such as *Fxr*^−/−^ and *Shp*^−/−^ mice, display enhanced *Ccnd1* expression^34,35^. AAV-FGF19-M52 lowered *Ccnd1* protein, as shown by immunohistochemical analysis, and transcript in aged Abcb4^−/−^ mice (Fig. 3c,d). Furthermore, dysregulated *cyclinE1* (*Ccne1*) expression, as well as *c-myc* gene, have been shown to act as potent oncogenes, and amplification of both genes promotes HCC formation^36,37^. *mRNA* analysis also revealed a marked inhibition of *Ccne1* and *c-myc* expression in AAV-FGF19-M52-injected *Abcb4*^−/−^ mice compared to controls (Fig. 3e).

### FGF19-M52 protects Fxr^−/−^ mice from HCC

**Figure 4:**
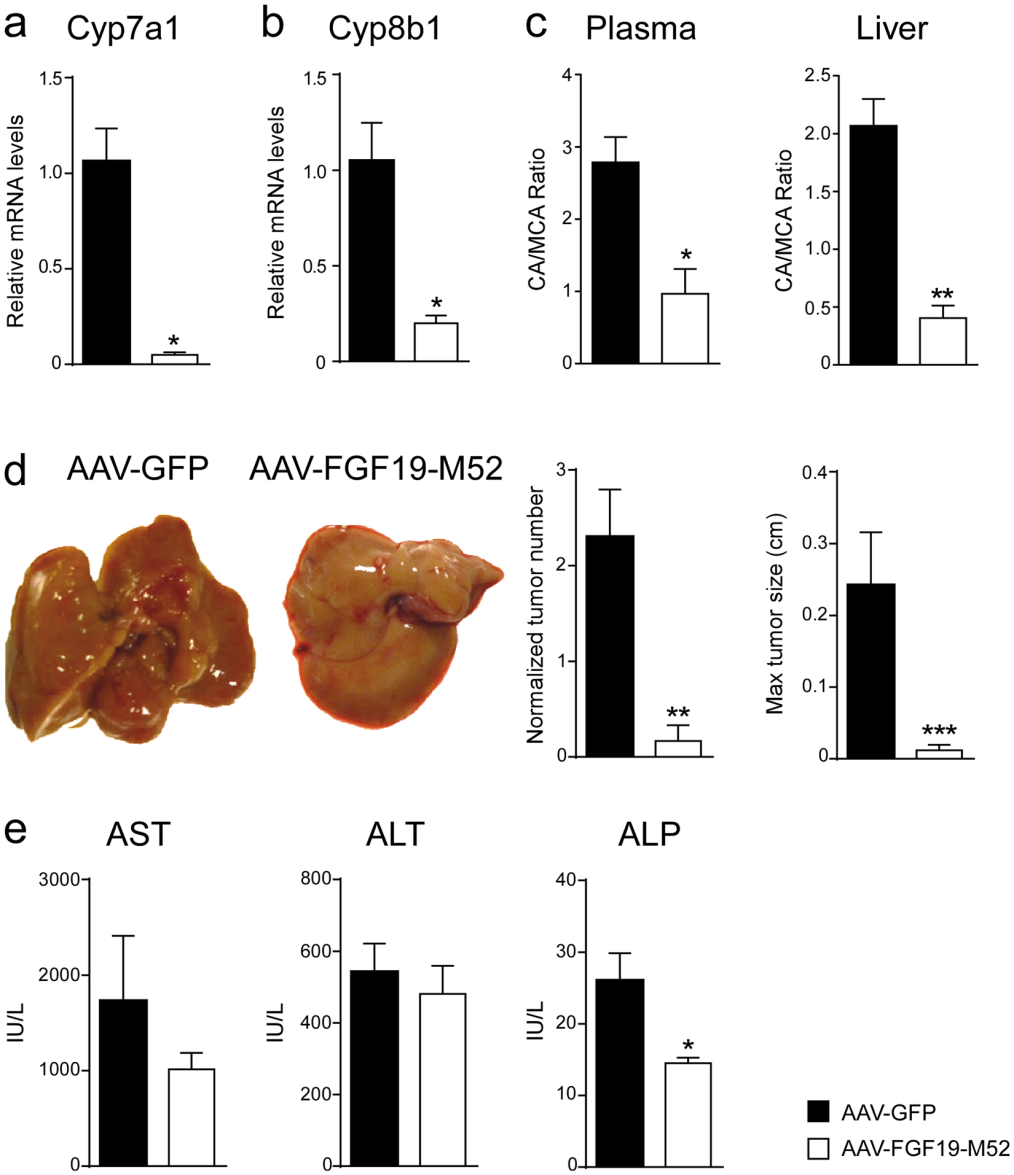
FGF19-M52 analogue protects Fxr^−/−^ mice from age-related liver damage and HCC progression. qPCR of hepatic (**a**) *Cyp7a1* (**b**) *Cyp8b1*. Expression was normalized to *Cyclophillin*. (**c**) BA composition (CA/MCA Ratio) in murine plasma and liver. (**d**) Macroscopic appearance of livers, normalized tumor number and maximum tumor size of 14-months-old *Fxr*^−/−^ mice (n = 8–16). (**e**) Biochemical parameters of liver damage in 14 months old *Fxr*^−/−^ mice. Statistical significance (*p < 0.05, **p < 0.01, ***p < 0.001) was assessed by Mann-Whitney’s U test.

**Figure 5:**
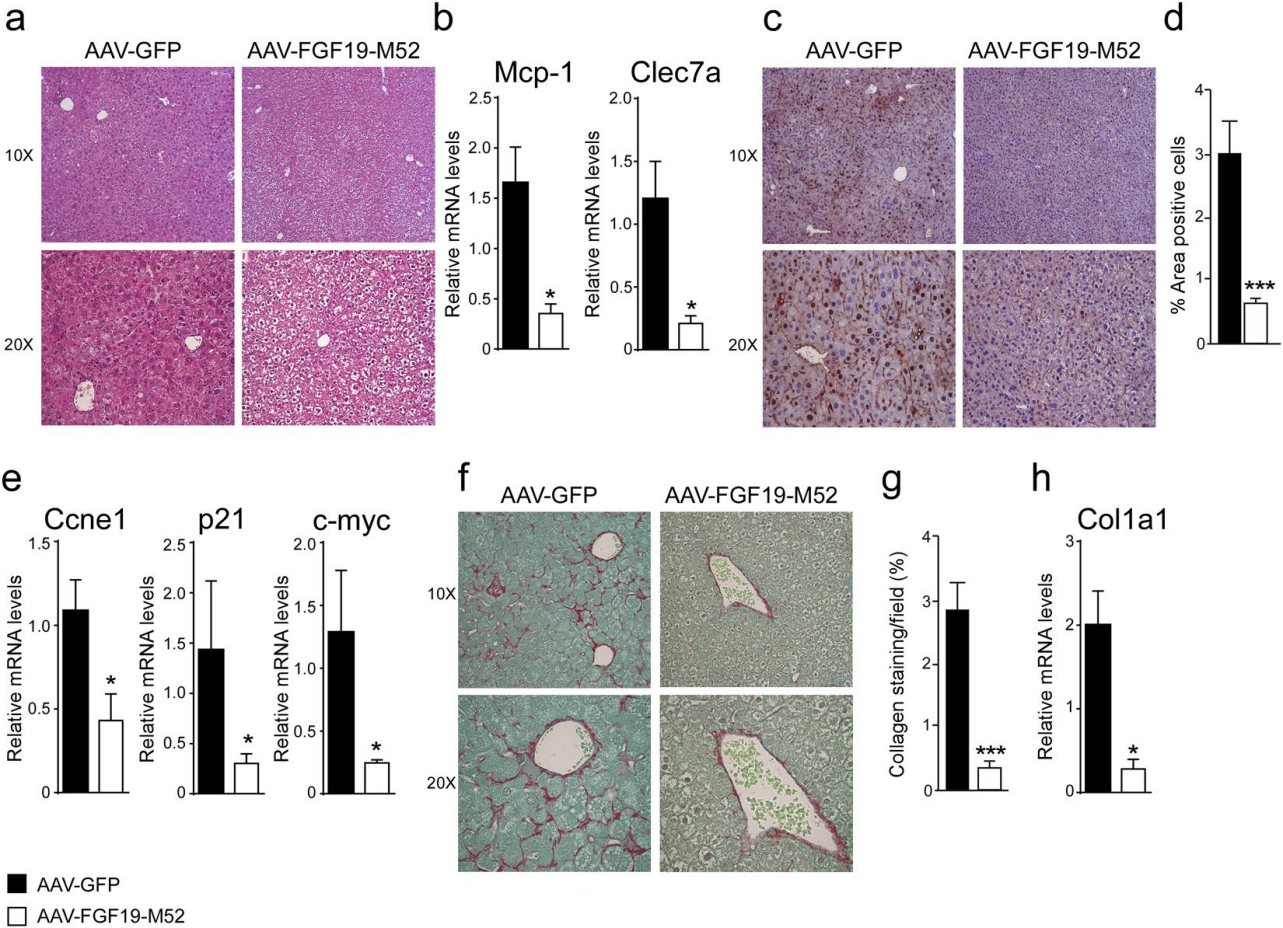
FGF19-M52 analogue protects Fxr^−/−^ mice from fibrosis and cellular proliferation. (**a**) Liver histology assessed by hematoxylin and eosin (H&E) staining and observed by light microscopy (magnification, 10–20X). (**b**) *Mcp-1* and *Clec7a* gene expression levels assessed by real time qPCR in tumor-free liver extracts. (**c**) Hepatic immunohistochemical staining of the oncogene Ccnd1. (**d**) Quantification of Ccnd1 staining assessed with ImageJ and expressed as % of the area occupied by positive cells. (**e**) *Ccne1*, *p21* and *c-myc* gene expression levels assessed by real time qPCR in tumor-free liver extracts. Expression was normalized to *Cyclophillin*. (**f**) Hepatic collagen deposition assessed by Sirius Red staining (magnification, 10–20X). (**g**) Quantification of collagen deposition assessed with ImageJ and expressed as % of collagen staining/field (**h**) *Col1a1* gene expression levels assessed by real time qPCR in tumor-free liver extracts. Expression was normalized to *Cyclophillin*. All values represent mean ± SEM. Statistical significance (**p < 0.01, ***p < 0.001) was assessed by Mann-Whitney’s U test.

We have previously shown that intestinal-specific Fxr reactivation, and in particular the entero-hepatic Fxr-Fgf15 axis activation, is able to prevent liver damage and its spontaneous hepatocarcinogenesis progression even in the absence of hepatic *Fxr*. In order to bypass the Fxr involvement and corroborate the importance of the intestinal hormone FGF19, we performed the same experiment and analysis conducted in *Abcb4*^−/−^ mice in aged *Fxr*^−/−^ mice. ELISA measurement shows a stricking amount of FGF19 in AAV-FGF19-M52-treated mice compared to AAV-GFP controls (AAV-GFP 0.33 ± 0.06 vs AAV-FGF19-M52 1021 ± 178.3 μM). AAV-FGF19-M52-injected mice displayed a significant lower hepatic *mRNA* expression of the key limiting enzymes of BA synthesis *Cyp7a1* and *Cyp8b1* compared to the control GFP-injected group (Fig. 4a,b). These changes were translated in a reduction of the plasmatic total BA pool size (AAV-GFP 11.48 ± 13.88 vs AAV-FGF19-M52 8.20 ± 3.59 μM) and a shift in both plasma and liver BA composition to a more hydrophilic BA pool profile due to the enrichment in MCA (Fig. 4c, Tables 1 and 2). 14 months old AAV-FGF19-M52 *Fxr*^−/−^ mice presented with a striking lower number of macroscopically visible liver tumors and smaller in size compared to controls (Fig. 4d). This was accompanied by a significantly decreased level of plasma ALP level and a lowered trend of ALT and AST compared to control mice (Fig. 4e). Liver morphology and HE staining shows liver injury and disrupted hepatic parenchymal structure in GFP-injected *Fxr*^−/−^ mice, in contrast with a more preserved hepatic parenchyma and less inflammatory infiltrates observed in FGF19-M52-treated mice (Fig. 5a). In parallel, a decrease in *Clec7a* and *MCP-1* was observed in FGF19-M52-injected mice compared to the control group (Fig. 5b). Previous studies have shown that, during aging, *Fxr*^−/−^ mice are characterized by enhanced fibrogenesis and cell proliferation^8,11,38,39^, therefore we performed Sirius Red and Ccnd1 stainings along with q-RTPCR analysis. M52-treatment revealed a protection in terms of fibrosis and collagen deposition at mRNA and protein level (Fig. 5f–h) as well as significantly lower hyperproliferative molecular status compared to GFP-treated controls as indicated by a decrease in protein and *mRNA* levels of *Ccne1*, *p21* and *c-myc* (Fig. 5c–e).

## Discussion

FGF19 is a post-prandial enterokine and a cornerstone of BA synthesis control, also regulating carbohydrate, lipid and energy homeostasis^40,41^. The landmark discovery of the FXR-FGF19 axis in the regulation of the BA homeostasis core^2,20,21,42^ opened new avenues for intestinal-specific therapeutic management of chronic diseases of the gut-liver axis. The therapeutic exploitation of the intestinal FXR/FGF19 axis activation in cholestasis^25^ and its translation into protection from HCC development in *Abcb4*^−/−^ mice^19^ prompt us to further examine the feasibility of a FGF19-based therapy in chronic liver and intestinal disease. However, literature data are debatable whether the enterokine FGF19 is per se implicated^22–24,26,28^ or not^43,44^ in HCC development. The in-depth characterization of FGF19 molecular structure and function allowed us to design a novel FGF19-based pre-clinical therapeutic agent uncoupling its metabolic activities from the proliferative ones. In fact, another novel engineered variant of FGF19 protein (M70) which fully retains BA regulatory activity but is devoid of murine pro-tumoral activity has been recently identified^45^. M70 differs from wild-type FGF19 by three amino acid substitutions (A30S, G31S, H33L) and a five amino acid deletion at the N terminus (P24-S28). Zouh *et al*. demonstrated that M70 interacts with the FGFR4 receptor and exhibits the pharmacologic characteristics of a biased ligand that selectively activates certain signalling pathways (e.g., cytochrome P450 7A1, phosphorylated extracellular signal–regulated kinase) and exclusion of others (e.g., tumorigenesis, phosphorylated signal transducer and activator of transcription 3)^45^. The identification of these types of FGF19 variants, including our M52, has greatly helped in overcoming the severe side effect of therapies targeting FGF19^46,47^, due to derangement in the gut-liver axis regulation of BA homeostasis and consequent development of liver toxicity and diarrhoea. The therapeutic potential of FGF19-M70 analogue has been well characterized and, unlike the natural form of FGF19, it does not show any pro-tumorigenic activity in 8 months old *Abcb4*^−/−^ mice, age at which these mice have not developed hepatic tumours yet^27^.

Here, we study the novel non-tumorigenic analogue FGF19-M52 as putative drug to inhibit *Cyp7a1*, reduce BA synthesis and eventually protect against BA-induced cancer in the liver. To this end, we show for the first time that the FGF19-M52 analogue protects against spontaneous hepatic tumorigenesis in 16 months old mice *Abcb4*^−/−^, through the reduction of BA concentrations and modification of the BA pool. Moreover, our results demonstrate that M52 retains control of BA negative feedback of the gut-liver loop and prevents BA-induced spontaneous hepatocarcinogenesis in 14 months old *Fxr*^−/−^ mice. Moreover, FGF19-M52 is also able to reduce *Cyp8b1* expression, while no changes in the expression of BA transporters (*Bsep*, *Ntcp*, *Oatp1* and *Oatp2*) and FGF19 receptors (*Fgfr4* and *β-klotho*) were found (data not shown), indicating that FGF19-M52 globally reduces BA levels without a direct transcriptional impact on their secretory or transport genes.

It is well known that both *Fxr* and *Abcb4* genes ablation in mice leads to liver damage, fibrosis, cholestasis and spontaneous HCC induced by high level of hydrophobic cytotoxic BAs. Indeed, the absence of *Abcb4* leads to accumulation of intraductal and biliary BAs that in absence of phosphatidylcholine are cytotoxic and exert their detergent activity that represent the *primum movens* for the *sequaela* of events that lead to HCC. On a different angle, the absence of *Fxr* leads to de-repression of hepatic Cyp7a1 with consequent potent increased BA synthesis and concentration systemically and within the liver. These events represent the leading step for liver damage, inflammation and fibrosis that bring *Fxr*^−/−^ mice to spontaneous HCC formation.

FGF19-M52-dependent inhibition of *Cyp7a1* and the consequent decrease of intrinsically harmful BA pool size and composition protect from hepatic tumour formation, even in the absence of Fxr. Mechanistically, the decrease of BA chronically high level and the shift of their composition into a more favourable hydrophilic one result in an outstanding inhibition of hepatic fibrosis that promptly translates into a blockage of overexpression of the typical HCC oncogenes *Ccnd1*, *c-myc* and others. Thus, in both models, FGF19-M52-dependent modulation of BA concentration with reduction of their levels and toxicity protect from liver damage and HCC.

Our findings support the concept that the control of BAs synthesis is definitely of great importance and could effectively reverse *Abcb4*- and *Fxr*-deficiency-associated hepatocarcinogenesis, suggesting that multiple metabolic players are involved in the hepatocarcinogenesis-preventing scenario. Our finding leverages the fine effort of testing novel FGF19 engineered variants that could display anti-fibrotic and anti-inflammatory effects but also antitumoral actions, thus opening *bona fide* a novel pharmacological strategy for example in PFIC patients who are susceptible to HCC formation even in young age.

## Methods

### AAV Production

AAV293 cells (Agilent Technologies) were cultured in Dulbecco’s Modified Eagle’s Medium (DMEM; Mediatech) supplemented with 10% fetal bovine serum and 1x antibiotic-antimycotic solution (Mediatech). Cells were transfected with three different plasmids (20 µg/plate, each) including AAV transgene plasmids, pHelper plasmid (Agilent Technologies) and AAV2/9 plasmid. Cells were harvested forty-eight hours after transfection. Viral particles in cell lysates were purified using a discontinued iodixanal gradient (Sigma-Aldrich). To determine the viral titer or genome copy number, viral stock was incubated in a solution containing 50 units/mL Benzonase, 50 mM Tris-HCl pH 7.5, 10 mM MgCl_2_, and 10 mM CaCl_2_ at 37 °C for 30 minutes. Viral DNA was cleaned with mini DNeasy Kit (Qiagen) and eluted with 40 µL of water. Viral genome copy was determined by quantitative PCR.

### Animal studies

For the characterization of the FGF19 analogue M52 obtained from NGM Biopharmaceuticals, Inc (se WO 2013/006486), 10–12 week old male *db/db* mice were purchased from Jackson Laboratory, and housed in a pathogen-free animal facility at 22ºC under controlled 12 hour light/12 hour dark cycle. All mice were kept on standard chow diet (Harlan Laboratories, Teklad 2918) and autoclaved water ad libitum. For *in vivo* tumorigenicity studies, cohorts *db/db* mice (n = 5) were randomized into treatment groups based on body weight. All animals received a single 200 μl intravenous injection of 3 × 10^11^ genome copies of either AAV-FGF19, AAV-M52, or a control virus encoding green fluorescent protein via tail vein. Animals were euthanized and livers were collected 24 weeks after injection of the AAV vectors for histology and gene expression analysis. These animal studies were approved by the Institutional Animal Care and Use Committee at NGM. For the HCC murine models, *Abcb4*^−/−^ mice on an FVB/N background were obtained from Charles River Laboratory (Charles River, Lecco, Italy) and whole-body *Fxr*^−/−^ mice were originally obtained from Dr. Frank J. Gonzalez (National Institutes of Health, Bethesda, MD). Male 8-weeks-old *Abcb4*^−/−^ and *Fxr*^−/−^ mice (n = 14–24 and n = 8–16, respectively) received a single intravenous dose of 1 × 10^11^ vector genome of AAV containing genes encoding either the FGF19-M52 form or control GFP. All mice were housed under a standard 12-hour light/dark cycle and fed standard rodent chow diet and autoclaved tap water ad libitum. Mice were aged until 14 and 16-months-old (*Fxr*^−/−^ and *Abcb4*^−/−^, respectively), sacrificed and analyzed for tumor development. All experiments were approved by the Italian Ministry of Health in accord with internationally accepted guidelines for animal care.

### Primary human hepatocytes

Primary hepatocytes from human livers (Life Technologies) were plated on collagen I-coated 96-well plates (Becton Dickinson) and incubated overnight in Williams’ E media supplemented with 100 nM dexamethasone and 0.25 mg/mL matrigel. Cells were treated with recombinant M52 or FGF19 proteins for six hours before lysis. Cells were treated with recombinant M52 or FGF19 proteins for six hours before lysis.

### Measurement of Plasma M52 and FGF19 Protein

Levels of human FGF19 and variants were measured in plasma using an ELISA assay (Biovendor). The assay recognizes both FGF19 and M52 in an indistinguishable manner.

### Blood Parameters

Levels of ALT, AST and ALP were measured with a colorimetric kit (BioQuant Heidelberg, Germany) according to manufacturer’s instructions.

### Chemicals

CA and other endogenous BAs were purchased from Sigma-Aldrich (St. Louis, MO). All solvents were of high purity and used without further purification. Acetonitrile for HPLC was from Merck (Darmstadt, Germany); methyl alcohol RPE, ammonia solution 30% RPE, glacial acetic acid RPE were from Carlo Erba Reagent (Milan, Italy); activated charcoal was from Sigma-Aldrich; and ISOLUTE C18 cartridges (500 mg, 6 ml) for the plasma sample pretreatment were purchased from StepBio (Bologna, Italy). Plasma BA free rat plasma was treated with activated 50 mg/ml charcoal and stirred at 4°C overnight. After centrifugation at 3000 g for 5 minutes the plasma was filtered through Millipore A10 Milli-Q Synthesis (0.45 µm) and stored at −20° C.

### Bile Acid Measurements

Plasma and hepatic BAs were identified and quantified by high-pressure liquid chromatography-electrospray-mass spectrometry/mass spectrometry (HPLC-ES-MS/MS) by optimized methods^48^ suitable for use in pure standard solution, plasma and liver samples after appropriate clean-up preanalytical procedures. Liquid chromatography analysis was performed using an Alliance HPLC system model 2695 from Waters combined with a triple quadruple mass spectrometer QUATTRO-LC (Micromass; Waters) using an electrospray interface. The analytical column was a Waters XSelect CSH Phenyl-hexyl column, 5 µm, 150 × 2.1 mm, protected by a self-guard column Waters XSelect CSH Phenyl- hexyl 5 µm, 10 × 2.1 mm. BAs were separated by elution gradient mode with a mobile phase composed of a mixture ammonium acetate buffer 15 mM, pH 8.0 (Solvent A) and acetonitrile:methanol = 75:25 v/v (Solvent B). Chromatograms were acquired using the mass spectrometer in multiple reaction monitoring mode. Plasma and hepatic bile acids were extracted using a standard, previously validated protocol^19^.

### mRNA extraction and quantitative real time qRT-PCR analysis

Total RNA was isolated from tumor-free livers using RNeasy Micro kit (Qiagen, Milano, Italy). cDNA was generated from 4 µg total RNA using High Capacity DNA Archive Kit (Applied Biosystem, Foster City, CA) and following the manufacturer’s instructions. mRNA expression levels were quantified by qRTPCR using Power Syber Green chemistry and normalized to cyclophillin mRNA levels. Relative quantification was performed using the ΔΔCT method. Validated primers for real time PCR are available upon request.

### Histology and Immunohistochemistry

Macroscopically tumor-free liver samples were fixed in 10% buffered formalin for 24 hours, dehydrated, and embedded in paraffin. Five-micrometer- thick sections were stained with hematoxylin-eosin (H&E) following standard protocols. Liver fibrosis was analyzed with Sirius Red by using Direct Red 80 and Fast Green FCF (Sigma Aldrich, Milan, Italy). Hepatocyte proliferation was assessed by immuno-histochemical detection of cyclin D1 (CCND1). All stained sections were analyzed through a light microscope. Histological features of hepatic disease have been assessed according to Histological Scoring System by Kleiner DE *et al*.^49^ and Brunt EM *et al*.^50^.

### Statistical Analysis

All measurements were performed in technical triplicates. All results are expressed as means ± standard error of the mean (SEM). Significant differences between three groups were determined by one-way ANOVA followed by Dunnett’s post hoc test, while between two groups were determined by Mann-Whitney’s U test. All statistical analyses were performed with GraphPad Prism software (v5.0; GraphPad Software Inc., San Diego, CA) and conducted as a two-sided alpha level of 0.05.

### Ethics Statement

The Ethical Committee of the University of Bari approved this experimental set-up, which also was certified by the Italian Ministry of Health in accordance with internationally accepted guidelines for animal care.
